# Integrated Evaluation of Urban Water Bodies for Pollution Abatement Based on Fuzzy Multicriteria Decision Approach

**DOI:** 10.1155/2015/327280

**Published:** 2015-09-30

**Authors:** Sarfraz Hashim, Xie Yuebo, Muhammad Saifullah, Ramila Nabi Jan, Adila Muhetaer

**Affiliations:** ^1^Department of Hydrology and Water Resources, Hohai University, Nanjing 210098, China; ^2^Department of Geotechnical, Civil Engineering, Hohai University, Nanjing 210098, China

## Abstract

Today's ecology is erected with miscellaneous framework. However, numerous sources deteriorate it, such as urban rivers that directly cause the environmental pollution. For chemical pollution abatement from urban water bodies, many techniques were introduced to rehabilitate the water quality of these water bodies. In this research, Bacterial Technology (BT) was applied to urban rivers escalating the necessity to control the water pollution in different places (Xuxi River (XXU); Gankeng River (GKS); Xia Zhang River (XZY); Fenghu and Song Yang Rivers (FSR); Jiu Haogang River (JHH)) in China. For data analysis, the physiochemical parameters such as temperature, chemical oxygen demand (COD), dissolved oxygen (DO), total phosphorus (TP), and ammonia nitrogen (NH_3_N) were determined before and after the treatment. Multicriteria Decision Making (MCDM) method was used for relative significance of different water quality on each station, based on fuzzy analytical hierarchy process (FAHP). The overall results revealed that the pollution is exceeding at “JHH” due to the limit of “COD” as critical water quality parameter and after treatment, an abrupt recovery of the rivers compared with the average improved efficiency of nutrients was 79%, 74%, 68%, and 70% of COD, DO, TP, and NH_3_N, respectively. The color of the river's water changed to its original form and aquatic living organism appeared with clear effluents from them.

## 1. Introduction

Massive ecosystem wide effects have been associated with their broad proliferation and toxin production [1]. The urban rivers or streams have always been the recipient of sewage water from various sources that have different kinds of the domestic, agricultural, and industrial foreign particles [2]. The odors released from these water bodies are offensive for environmental pollution; the release of odors has been often unavoidable due to its natural phenomena [3]. The municipal sewage is the mixture of various organic matters, and the decomposition of these matters produces harmful gases that in fact deteriorate the environment and logically created the infection diseases [4]. The inherent expectation is that villagers under this sewer situation have been leaving their houses because they face polluted potable water and skin and waterborne infectious diseases, and therefore urban environment situation has become more alarming [5, 6]. Municipal wastewater is the main cause of environmental impact, if it is directly discharged into urban water bodies or rivers without any sanitation preliminary treatment. Moreover, due to an increase in the shortage of clean water, there is a need for convenient management of accessible water resources.

To control environmental pollution would be a huge challenge for the planner and policy maker, due to treatment cost as well as the unbridled population acceleration. Here we need solution that efficiently resolves these critical pollution problems and could be used to rehabilitate the existing systems. In the last decades, some conventional technologies and methods have been developed and applied. To begin the application of reaeration (traditional technology) as adopting a series of weirs [2, 7], moving the wastage discharges place and local oxygenator were used for pumping air into the water body for wastewater treatment [8, 9]. The application of Multifunctional Constructed Wetland has been used for the treatment of wastewater and the purification of river water [10–12]. Nitrogen and some other nutrient wastes were the vital source of pollution problems in China and unable to restore system through these conventional techniques. So, the anaerobic ammonia oxidation researches tend to move into full-scale treatment plant. Till now at least 5 genera and 13 species have been identified using culture-independent-molecular techniques [13]. Within the last decades some specialized reactor systems such as sequencing batch reactor, rotating biological contactor, trickling filter, UBF reactor, granular sludge bed reactor, and membrane bioreactor have been introduced in both laboratory and full scale to obtain high removal rate, and finally we noticed that these reactors played an important role in securing high rate performance for the product of nitrogen removal. Nitrogen pollution causes serious environmental problems and it not only threatens the sustainable development of fisheries, agriculture, tourism, and so forth, but also is harmful to the living environment of human beings. The amounts of phosphorus and ammonia nitrogen in domestic wastewater have been noticed from 10 to 17 and 30 to 50 mg/L, respectively [14].

Ecofriendly and sustainable environmental demand is the hot and impressive topic due to public, economic, and legislation pressure. The best selection in complex framework is based on the sustainability of nutrient removal, biodegradation of suspended particles, and removal efficiency of the system. In this situation, the major preference is to adopt any system that is more reliable for energy consumptions, conversion of chemicals into biomass, complex infrastructure, and the repairing or maintenance cost of the system. Bacterial Technology (BT) provides a plenty of opportunities for effectively treating these issues [2]. BT is an application of bioremediation that uses microorganism metabolism to remove nutrients from the water bodies and regenerate up to the original condition [2, 6, 9], and its operational cost is relatively low [15], which generally have a high public interest. Due to its smart application, it is popular in the research area of environmental sciences and engineering. Temperature is the major concern that is directly effective in the process of the degradation of the substances [9].

Due to these considerations, we can adopt the new technique as BT with complete confidence. Their bacteria are usually hired to vitiate pollutants or nutrients into simple or nontoxic entity and produce suitable effluents [16]. This technology has reassuring advantages compared to other traditional techniques as already discussed. BT has been successfully implemented to recover the polluted lakes [17], restore polluted rivers, and assimilate effluent of wastewater treatment plant [9]. To control the urban river pollution, BT has been employed in different places in China, that is, for treating the polluted urban water bodies [2, 15]. It was determined to be successful with reliable results in boosting up the recovery processes of all water bodies compared to the other traditional technologies. It extends for the rehabilitation of polluted lakes, rivers, and streams and is also reliable for the requirement of the wastewater effluent standards without constructing massive structures as compared to the other conventional methods [15, 16].

In the past, the water quality index approach was considered as the best tool to determine the water quality of the water bodies [17, 18]. Numerous researches represent the integrated uncertainty in evaluating the water quality. Some of these are under the base on fuzzy impartial optimization [19, 20] and MCDM problems using AHP [21]. Further, a few years ago, AHP and FAHP were acquiring popularity in the hydraulics and environmental engineering fields [22]. Srdjevic and Medeiros [23] have used FAHP for the management plans and Singh et al. [19] have used FAHP to determine the water quality of the Yamuna River that is tributary of Ganga. In this research, the urban rivers pollution abatement is being extensively determined at five various places, such as Xuxi River, Wuxi City; Gankeng River, Shenzhen City; Xia Zhang River, Yixing City; Fenghu and Song Yang Rivers, Ruian City; Jiu Haogang River, Hangzhou City. A location-wise variation of the water quality parameters (temperature, DO, COD, TP, and NH_3_N) was determined before and after the treatment of the BT. For the relative significance of water quality parameters, AHP has been applied in the selected sites. In addition, FAHP is developed for the present research to determine the original status of water quality on each urban river based on MCDM framework. For current study, the data matrix is very complicated, because the spatial and temporal parameters vary from site to site. It is not possible with simple AHP to evaluate the pollution status on each station of each site. Therefore, FAHP is the best technique which can help to determine the water quality parameters values as compared to the other techniques. In this paper we briefly discuss practical implementation of BT on urban polluted rivers and argue with MCDM based on FAHP that BT is simple, affordable, and sustainable for restoring polluted water bodies.

## 2. Materials and Methods

### 2.1. Study Area and Samples Collection

Xuxi River (XXR) is situated in Wuxi City, Chang Nan District of China, geographically as (31°56.29′N and 120°28.14′E). Its upper stream starts from the Jing-Hang main canal and travels towards the ancient small canal. The selected river length for the experiment is 1360 m with 4.5 m of upstream surface average width and about the average depth of 1.4 m. River is under north subtropical humid zone and is marked by muddy sediments. This zone is facing four distinct seasons with the phenomenon of climatic influence circulation. Fenghu (FH) and Song Yong (SY) Rivers were selected, FH River is placed on the tail of the SY River, and both of them are situated in Wenzhou (Rui'an) City (120°39.13′E and 27°46.49′N), Zhejiang Province, China. Most of the Wenzhou area is placed under the typhoon zone, and the FH River is taking water from Wenruitang and Liangmian Rivers. The SY River is starting from cave bridge and falls directly into the FH River. The SY River length is about 280 m with the average breadth 5–18 m and 1–3 m water depth, and FH river length is about 740 m with the average breadth 6–15 m and 1 m water depth. For monitoring and sample collection of the experiment, the selected reaches of both rivers were divided into six points. The appearance of the river water color was blackish or greenish, and bubbles were blowing on the surface of water. These rivers are situated under the commercial and highly polluted area, and almost 2000 m^3^ sewage water enters into them [2]. Therefore, the average depth of sediments is 0.1 m and the river's water quality was unsuitable for any purpose.

The remaining sites of Gankeng River (22°33.24′N and 114°34.63′E), Xia Zhang River (31°26.49′N and 119°49.13′E), and Jiu Haogang River (30°18.59′N and 120°09.07′E) are placed in Shenzhen City, Yixing City, and Hangzhou City of China. The averaged physiographic conditions of these rivers are the same as the above rivers. On the basis of Chinese surface water quality standard, the rank or class of water quality in the source section was determined to be grade V (Table 1). Class V shows the worst (poor) by the Chinese National Standard board and least water quality standard. For any purposes, this water quality of the river is extremely unsuitable. Hence, these sites selected for small urban rivers belong to the worst Class V category by the Chinese National Standard (CNS) board.

### 2.2. Bacterial Implementation

Bacterial Technology (BT) is applied in a simple way, and its procedure is held under three kinds of material as Bacterial Clusterization (BC), Nature Liquid, and Biological Filter Media (4 : 3 : 3). BC is an important material that has a mixture of three types of ingredients as beneficial bacteria (bacilli,* Bacteroides*, brown-rot spindle, and Lactobacillales, denitrifying with 6 : 4 : 3 : 4 : 3), mix medium (catalyst process as glucose, sucrose, cellulose liquid, yeast cream, liquorice root, magnesium sulfate, potassium hydride, mannitol, tartaric acid (Na; K), folic acid, and ammonium nitrate), and water [24]. The mixing ratio represents that it is harmless and has no any adverse effects. Nature Liquid (NL) is the mixture of trace element, multiple enzymes, humic acid, amino acid, and vitamins and composition of each adequate substance on judgment. Biological Filter Media are used on a domestic level as the gap string filter media.

**Figure 1:**
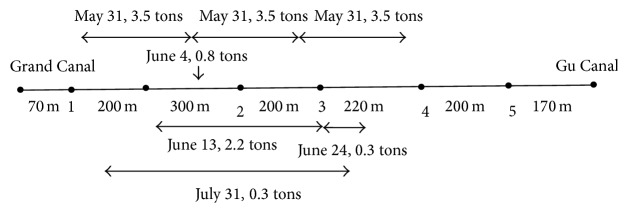
Schematic diagram of the Xuxi River and sampling points during BT.

By implementation of BT on site, the bacterial amount as BC is added to the selected points of each river as shown in Figure 1 (example on XXU site). As the bacterial agent is used to effectively work under relatively constant and slow flow of velocity, an artificial weir is installed at the end of the river reach which is the small wood bridge to stop effluent. It was technically built at about 50 cm high above the water surface level in order to extend the hydraulic retention time. This experiment was conducted from May 31 to July 31. To employ BT operation, the implementation procedure could vary based on the physical condition of the site. However, the method of adding beneficial bacteria directly to the polluted water body has proven to achieve desirable results for restoration programs. The addition of beneficial bacteria to polluted river is usually termed the Bacterial Technology.

### 2.3. Samples Collection Procedure

The sampling network was managed to cover the complete range along the inlet and outlet points of the rivers and determined the dominant point sources that have an impact on the water quality. Both of the sites are located under the area of population and industrialization, so the samples were collected from various depths (0.5 ft and >1.5 ft), at each monitoring station. The samples were collected from 8:30 AM to 4:30 PM during the period of experiment and 5 to 8 times in each month. To evaluate the water quality, the samples were kept in polyethylene bottles and stored in insulated ice cooler that were delivered to the laboratory on the same day. All the samples were saved at 4°C until the analysis and processing.

## 3. Numerical Calculations for Data Treatment

All mathematical and statistical calculation was analyzed by using Excel 2007 and MATLAB Fuzzy Logic Function. There have been various methods on Multiattribute Decision Making (MADM) and the most useful is AHP which especially is based on pairwise comparisons on a ratio scale [25]. According to some AHP limitations the fuzzy modification of AHP (FAHP) was then posed that is the subject of this study.

### 3.1. Analytical Hierarchy Process (AHP)

**Figure 2:**
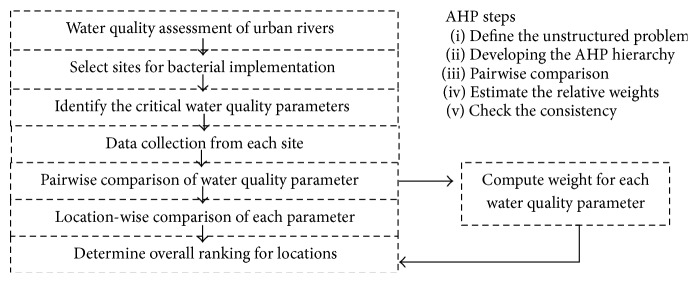
AHP for judgment.

AHP is an MCDM method that provides the hierarchical framework to illustrate the concern objective and developed the scale of priority based on the application judgment [25]. The AHP operation belongs to six essential steps [26] as shown in Figure 2.

#### 3.1.1. Define the Unstructured Problem

We define the concern objectives and consequence of the unstructured problem and the recognition of the specific characteristics.

#### 3.1.2. Developing the AHP Hierarchy

**Figure 3:**
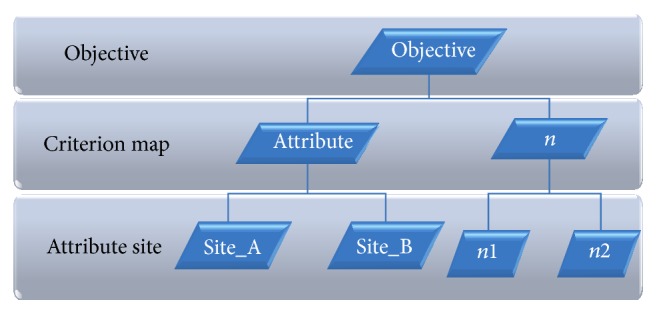
Hierarchical structure of decision problem.

The AHP is based on the decision disintegration of the hierarchy unstructured problem that resides in the decision problem of the most important element [27]. The complicated task is decomposed into a hierarchical structure (Figure 3) with the elements of decision.

#### 3.1.3. Pairwise Comparison

For pairwise comparison matrices of each element of the hierarchy structure are compared as follows:

(1) $$\begin{array}{c} A=\left[\begin{array}{cccc} 1 & \frac{w_{1}}{w_{2}} & \cdots & \frac{w_{1}}{w_{n}}\\ \frac{w_{2}}{w_{1}} & 1 & \cdots & \frac{w_{2}}{w_{n}}\\ \vdots & \vdots & \vdots & \vdots \\ \frac{w_{n}}{w_{1}} & \frac{w_{n}}{w_{2}} & \cdots & 1 \end{array}\right], \end{array}$$

where *A* is matrix of pairwise comparison, *w*_1_ is element weight 1, *w*_2_ is element weight 2, and *w*_*n*_ is element weight *n*.

For the decision of the relative significance between hierarchy elements in matrix *A*, a linguistic scale is employed for the values to be rated from 1 to 9 (Table 2).

#### 3.1.4. Estimate the Relative Weights

The relative weights of elements in each pairwise comparison matrix are determined by some methods like eigenvalue method. The relative weights (*W*) of matrix *A* are determined as

(2) $$\begin{array}{c} \left(\;A-\lambda \max I\;\right)\times \omega =0, \end{array}$$

where *λ*max is matrix *A* as biggest eigenvalue and *I* is unit matrix.

#### 3.1.5. Check the Consistency

The matrices consistency property is determined to ensure that the judgments of decision makers either are consistent or need more iterations. Consistency Index (CI) can be measured from the following equation:

(3) $$\begin{array}{c} CI=\frac{\lambda \;max-n}{n-1}. \end{array}$$

The reciprocal matrix is generated from the random Consistency Index that would be known as the random index (RI). A sample size of 100 was used to generate the average RI for the matrices of order of 1–15 [28]. The Saaty matrices represent the RI (Table 3) that can be seen in the order of 1–10 [25]. At last, if CR < 0.1, the judgments from the above procedure are consistent and the derived element's weights can be considered for the further analysis. The formulation of CR is

(4) $$\begin{array}{c} CR=\frac{CI}{RI}. \end{array}$$

#### 3.1.6. Obtain the Overall Rating

At the end, the relative decisions of element weights are compiled to gain the whole alternatives rating as follows:

(5) $$\begin{array}{c} {w_{i}}^{s}=\overset{j=m}{\underset{j=1}{\sum }}{w_{ij}}^{s}{w_{j}}^{a}\;i=1,\ldots ,n, \end{array}$$

where *w*_*i*_^*s*^ is total weight of “*i*” site, *w*_*ij*_^*s*^ is weight of alternative *i* associated with attribute *j*, *w*_*j*_^*a*^ is weight of attribute *j*, *n* is number of sites, and *m* is number of attributes.

### 3.2. Fuzzy Analytical Hierarchy Process (FAHP)

**Figure 4:**
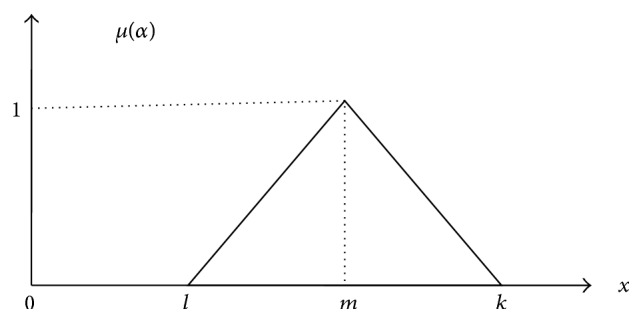
Fuzzy triangular Number.

Despite the recognition of AHP often this method is censured to sufficiently handle its failure for the imprecision and latent uncertainty associated with the grading of the decision maker's perception of exact values [29]. Fuzzy AHP as an extension of AHP investigate to be more efficient tool in the water management decision problems [30, 31]. Since vagueness and fuzziness are ordinary characteristics in a number of decisions, a FAHP method should be able to indulge ambiguity or vagueness [32]. In FAHP, the eigenvector method is applied to simulate the reciprocal matrix and to evaluate the importance and alternative performance across the criteria. The additive weighting method is applied for the determination of the use of alternative across criteria. When complex multifeatures are considered for decision making problems, FAHP has skill of capturing an uncertainty of human assessment [33]. This procedure is applied to determine the crisp judgments into fuzzy judgments [34]. This classic fuzzy set theory allowed [0,1] range of real numbers to operate the participation functions. The major fuzziness function is the individuals grouping elements into classes without clearly defining the boundaries [35]. The uncertainty judgment of comparison can be indicated by the fuzzy number. A fuzzy number of the triangle is defined by three real numbers (Figure 4) which belong to special class, expressed as (*x*, *b*, *k*). The fuzzy numbers of the triangle are determined as follows:

(6) $$\begin{array}{c} \mu \left(\;\alpha \;\right)=\left\{\begin{array}{cc} \left(x-l\right)\left(m-l\right), & l\leq x\leq m,\\ \left(k-x\right)\left(k-m\right), & m\leq x\leq k,\\ 0, & \text{otherwise.} \end{array}\right. \end{array}$$

In order to compose pairwise alternatives comparison under each criterion or benchmark, a triangular fuzzy comparison matrix is indicated as follows:

(7) $$\begin{array}{c} \left[\begin{array}{cccc} \left(1,1,1\right) & \left(l_{12},m_{12},k_{12}\right) & \cdots & \left(l_{1n},m_{1n},k_{1n}\right)\\ \left(l_{21},m_{21},k_{21}\right) & \left(1,1,1\right) & \cdots & \left(l_{2n},m_{2n},k_{2n}\right)\\ \vdots & \vdots & \vdots & \vdots \\ \left(l_{n1},m_{n1},k_{n1}\right) & \left(l_{n2},m_{n2},k_{n2}\right) & \cdots & \left(1,1,1\right) \end{array}\right], \end{array}$$

where ${\tilde{\alpha }}_{ij}=\left(l_{ij},m_{ij},k_{ij}\right)$; ${{\tilde{\alpha }}_{ij}}^{-1}=\left(l_{ji},m_{ji},k_{ji}\right)$, for *i*, *j* = 1,…, *n* and *i* ≠ *j*.

Total alternatives preferences and weights can be acquired from different method. In this study, these two approaches or techniques will be posed in renewal.

#### 3.2.1. Fuzzy Logic Process for Experiment

A fuzzy analytical hierarchy process (FAHP) has been developed to evaluate the status of water quality at the selected stations along each river under a Multicriteria Decision Making framework. A decision support mechanism has been introduced to select and prioritize stations, with specific reference to the universal principle as written below:

- Moving water tends to contain more DO than stagnant water.
- The DO concentration is inversely proportional to temperature.
- Health of water quality is based on the requirement of organism that lives in it.
- pH scale verifies the acidity and alkalinity of wastewater.
- The overenrichment of a body of water by nutrients like nitrates and phosphates is cause of eutrophication.

The various water quality parameters have been considered as criteria to evaluate water quality status at a given station of each project site. Pairwise comparisons of the criteria and the stations have been performed to assess water quality using linguistic variables.

## 4. Result and Discussion

The selected urban rivers are situated under the appalling environment and the river's conditions were awful before the operation of BT. The huge amount of sewage was loaded directly and entered into these rivers. In addition, it is observed that there was not any preliminary facility to control or dump the domestic sewage. Therefore, the sewage is partially or directly a part of the urban river without any pretreatment. Under this alarming situation, the river's color was changed into greenish representing the thick oil floats and debris. Therefore, in this sewer condition, any living organism in the river water could not exist.

**Figure 5:**
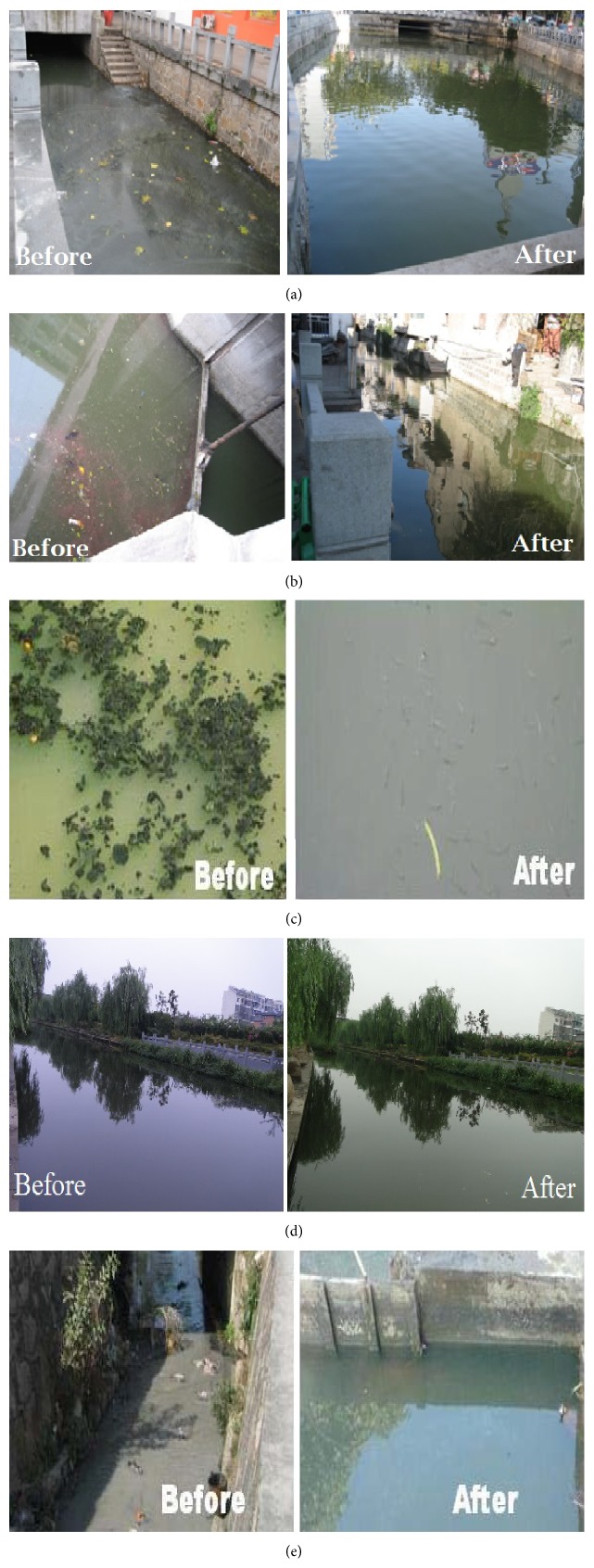
(a) Contrast diagram of Fenghu and Song Yang Rivers. (b) Contrast diagram of Xia Zhang River. (c) Contrast diagram of Xuxi River. (d) Contrast diagram of Jin Haogang River. (e) Contrast diagram of Gankeng River.

BT has been applied and water samples from the selected points were collected before and after the treatment of the experiment. The physiochemical parameters were collected on the specific monitoring points on every site. The range, mean, and standard values of each parameter are in Table 4; after determining the values, we compared the improved efficiency of nutrients before and after bacterial action which was 79%, 74%, 68%, and 70% of DO, COD, TP, and NH_3_N, respectively. From the results, the DO was the most critical parameter for aquatic life of rivers that have maximum efficiency. To protect the environmental pollution, we determined that TP and COD efficiency also have favorable results. For more consideration, the color and algal from every site are also recovered as shown in Figures 5(a)–5(e).

### 4.1. Numerical Evaluation of the Experiment

The fundamental statistics of these restoration experiments are based on 2760 total water samples (23 sampling stations × 4 sampling frequencies × 5 replications × 6 months) and are summarized in Table 4 which represents the range, mean, and standard deviation of the results for each parameter. The data were collected during 6 months and each station of the site was monitored with spatial as well as temporal variation.

FAHP developed a selection support tool that describes the pairwise priority of the station with the particular beneficial reference, such as domestic, aquatic status, irrigation, and recreational and industrial enterprises. The pairwise comparison matrix was formulated due to the variations and the complicity of the water quality parameters on each site, and the comparisons were accomplished based on the convincing of engineering results and each water quality parameter of all sites was formulated in Table 5.

For the evaluation of the relative weights, the comparisons of all five locations with each parameter (Tables 6 [Table tab7] [Table tab8] [Table tab9]–10) were measured with the ambition to determine the actual status of the water quality improvement from before and after the BT operation.

**Figure 6:**
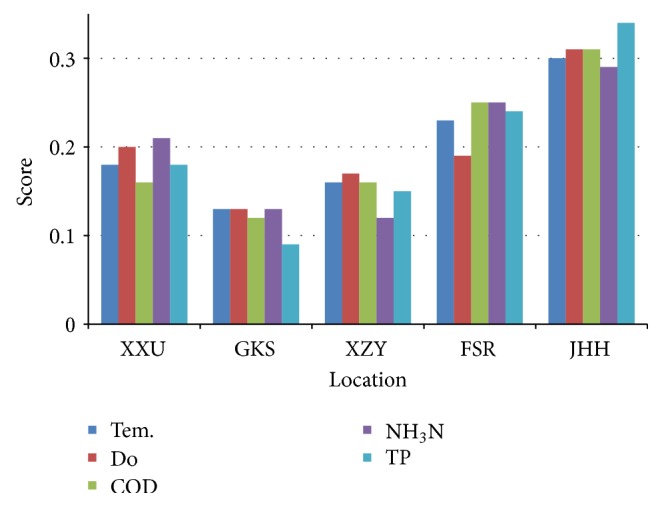
Ranking criteria of each water quality parameter in each location.

The main concern in this present contribution is to explain the actual BT function to mitigate the pollution from urban water bodies. AHP based on FAHP results are applied to the ranking of the water quality parameters (Table 11) as well as the location ranking with an overall inconsistency of 0.076 (Table 12). The location-wise variations score is illustrated in Figure 6, which represent the high ranking of the JHH site as compared to the others.

After the evaluation of the current status from the results, the actual pollution status of each site after the BT operation is revealed. Figure 6 displays the summary of the results with the overall inconsistency of 0.076, less than 10%. Its describe the COD value is extremely high (up to 139 mg/L) at JHH site which display the major cause of pollution. Besides, the river is placed in the industrial area. Similarly the value of TP is also high because, in the middle, a cement factory is working and the wastewater directly enters into the river. Instead of all, for the evaluation of BT, in the beginning of operation, there was an appalling condition as blackish water and odors that made part of the pollution. When we applied BT operation, the polluted river was changed up to reliable condition without any odors. The river water color was also changed from blackish to its original form. BT restored the JHH River and it came back to its habitat environment. However, we can adopt these advanced technologies to rehabilitate our environment, but there is a need to manage or fix horrific point sources of pollution.

### 4.2. Cost Benefit Ratio Based on Conventional Technologies

Temperature is the major concern under the metabolism process of the bacteria, with higher temperature during the summer (20–30°C) and lower values in the winter season (10–18°C). The plus advantage of BT does not require destruction of an already built system. BT has no effect on the natural environment because it does not involve the use of chemicals. Therefore, it is helpful for friendly ecology. It is free from all other issues, as high construction and maintenance costs can be a huge burden to organization and policy makers.

In view of this, with revolutionary calculations, the adoption of BT has been concluded to be the most convenient approach for developing countries [15]. The cost to treat the tons of wastewater is about 241≈321$, where WWTPs are being built, under construction, or already built, and the operation cost is between 0.12 and 0.22$ per ton. According to this statement, for the municipal sewerage operation system needs to spend above 16 × 10^7^$ on single attempt. If the pipe network of municipal administration is built, the amount of total cost will exceed 32 × 10^7^$, and the operational cost is raised up to 4 × 10^7^$ annually. Therefore, with the addition of bacteria to treat the sewerage wastewater, the one-off investment cost is only 65$ and this method is simple, easy to operate, and affordable. For the long term, BT maintenance and artificial dregs cannot be needed for 10 years in the future [36]. In addition, the existing sanitation systems are deteriorating due to many-imperfection care. So BT has ability to restore these systems due to its self-purification property. Similarly the maintenance cost of the sewerage system is unfavorable due to economic collapse. So we can prefer this technology due to its simplicity and low cost.

## 5. Conclusions

In order to rehabilitate the urbanized water bodies as lakes, rivers, and streams, BT is sustainable and reliable for public health with no maintenance and further general costs to minimize the traditional system. In this study, we demonstrate the interpretation of the water pollution problems of a complex dataset through MCDM techniques, because chemometric research enables us to discuss the similarities and dissimilarities along the observing stations among the variables that could not be clearly visible for assessment of the analytical data in a table. This research emphasized that the BT offers an ingenious and innovative solution for rehabilitation of the urban water bodies up to the suitable water quality. BT is efficient due to its simplicity, being economically affordable and reproducible on any scale of the operation; hence, it can provide tenable and long-term solution to the various water related pollution problems all over the world.

## Figures and Tables

**Table 1:** Water quality parameters before BT and Chinese National Standard.

Sampling time		Monitoring project						
		Water temperature °C	pH	DO mg/L	COD mg/L	TP mg/L	TN mg/L	NH_3_N mg/L
National standard GB3838-2002		Class V index	6–9	2.00	15.00	0.0	2.00	2.00
A	14:40	16.1	7.5	2.5	10.90	0.96	14.80	10.60
	River water quality class		—	V	V	Inferior V	Inferior V	Inferior V
B	16:00	27.2	8.77	2.81	12.10	0.82	14.90	11.20
	River water quality class		—	V	V	Inferior V	Inferior V	Inferior V

^*∗*^A, B represent two criteria based on pH and temperature value.

**Table 2:** Scales for pairwise comparison [25].

1	Equal importance
3	Moderate importance
5	Strong importance
7	Very strong importance
9	Extreme importance
2, 4, 6, 8	Intermediate values between adjacent scale values

**Table 3:** Random inconsistency indices [25].

Number of criteria	1	2	3	4	5	6	7	8	9	10

RI	0	0	0.58	0.9	1.12	1.24	1.32	1.41	1.45	1.49

**Table 4:** Water quality data of experiment.

Parameters	GB2828-2002 (CNS)		XXU	GKS	XZY	FSR	JHH
Tem. (°C)	15–30	Mean Std. deviation Minimum Maximum	24.7 0.29 19.7 27.8	25.5 0.46 24.6 25.8	16.5 0.32 16.2 17.1	16.2 0.34 15.6 16.8	28.7 0.35 27.9 29.4

DO (mg/L)	2	Mean Std. deviation Minimum Maximum	1.69 1.08 0.4 3.3	1.79 0.9 0.4 2.7	1.83 1.9 0.5 4.8	1.14 0.7 0.8 1.8	3.4 1.38 0.7 4.5

COD (mg/L)	15	Mean Std. deviation Minimum Maximum	14.5 6.8 5.4 17.7	16.4 2.45 4.1 18.1	59.7 17.5 29 67.2	43.5 23.5 24.7 86	59.01 35.2 24 139

NH_3_N (mg/L)	2	Mean Std. deviation Minimum Maximum	15.21 9.77 9.42 27.3	16.87 10.23 8.01 26.9	22.49 9.99 12.04 29	13.59 7.5 0.87 15.7	21.44 10.44 8.76 26.7

TP (mg/L)	0	Mean Std. deviation Minimum Maximum	0.91 0.57 0.14 1.86	1.2 0.5 0.18 1.57	0.83 0.49 0.07 1.2	1.3 0.47 0.5 1.7	1.9 0.42 0.7 2.8

**Table 5:** Pairwise comparison matrix of the various water quality parameters.

Parameters	DO	COD	TP	NH_3_N	Temperature
DO	1	1/3	1	1/1.5	1/5
COD	3	1	2	1/1.5	1
TP	1	1/2	1	1/2	1
NH_3_N	1.5	1/2	2	1	1/1.5
Temperature	5	1	1.5	1.5	1

**Table 6:** Location-wise comparison matrix for temperature.

Site	XXU	GKS	XZY	FSR	JHH
XXU	1.00	0.33	0.67	2.00	0.29
GKS	3.00	1.00	1.49	4.00	0.67
XZY	1.50	0.67	1.00	2.00	1.00
FSR	0.50	0.25	0.50	1.00	0.33
JHH	3.50	1.50	3.00	3.00	1.00

**Table 7 tab7:** Location-wise comparison matrix for DO.

Site	XXU	GKS	XZY	FSR	JHH
XXU	1.00	1.50	0.33	0.29	0.57
GKS	0.67	1.00	0.29	0.50	1.00
XZY	3.00	3.50	1.00	4.00	1.00
FSR	3.50	2.00	0.25	1.00	3.00
JHH	1.75	1.00	0.33	0.33	1.00

**Table 8 tab8:** Location-wise comparison matrix for COD.

Site	XXU	GKS	XZY	FSR	JHH
XXU	1.00	2.00	4.00	0.80	4.00
GKS	0.50	1.00	0.33	0.59	4.00
XZY	0.25	3.00	1.00	0.67	1.00
FSR	1.25	1.70	1.50	1.00	2.00
JHH	0.25	0.25	0.25	0.50	1.00

**Table 9 tab9:** Location-wise comparison matrix for NH_3_N.

Site	XXU	GKS	XZY	FSR	JHH
XXU	1.00	0.80	0.40	1.33	0.67
GKS	1.25	1.00	0.67	2.00	0.33
XZY	2.50	1.50	1.00	9.09	1.00
FSR	0.75	0.50	0.11	1.00	0.40
JHH	1.50	3.00	0.40	2.50	1.00

**Table 10:** Location-wise comparison matrix for TP.

Site	XXU	GKS	XZY	FSR	JHH
XXU	1.00	4.00	1.49	0.67	0.20
GKS	0.25	1.00	0.67	0.29	0.33
XZY	0.67	1.50	1.00	0.67	1.00
FSR	1.50	3.50	1.50	1.00	0.67
JHH	5.00	3.00	0.25	1.50	1.00

**Table 11:** Criteria ranking of water quality parameters.

Parameters	Scores	Ranking
Temperature (°C)	0.305	1
COD (mg/L)	0.277	2
DO (mg/L)	0.204	3
TP (mg/L)	0.116	4
NH_3_N (mg/L)	0.097	5

**Table 12:** Criteria ranking of sites (overall inconsistency = 0.076).

Site	Scores	Ranking
JHH	0.310	1
FSR	0.241	2
XXU	0.191	3
XZY	0.175	4
GKS	0.083	5
